# Supplementary material for: Intracellular Localization in Zebrafish Muscle and Conserved Sequence Features Suggest Roles for Gelatinase A Moonlighting in Sarcomere Maintenance
Source: Biomedicines. 2019 Nov 29;7(4):93. doi: 10.3390/biomedicines7040093 (PMC6966518; doi:10.3390/biomedicines7040093)
Supplement: Supplementary file 1 [file biomedicines-07-00093-s001.pdf]

biomedicines

Article

Supplementary Material: Intracellular Localization in Zebrafish Muscle and Conserved Sequence Features Suggest Roles for Gelatinase A Moonlighting in Sarcomere Maintenance

Amina M. Fallata, Rachael A. Wyatt, Julie M. Levesque, Antoine Dufour, Christopher M. Overall and Bryan D. Crawford

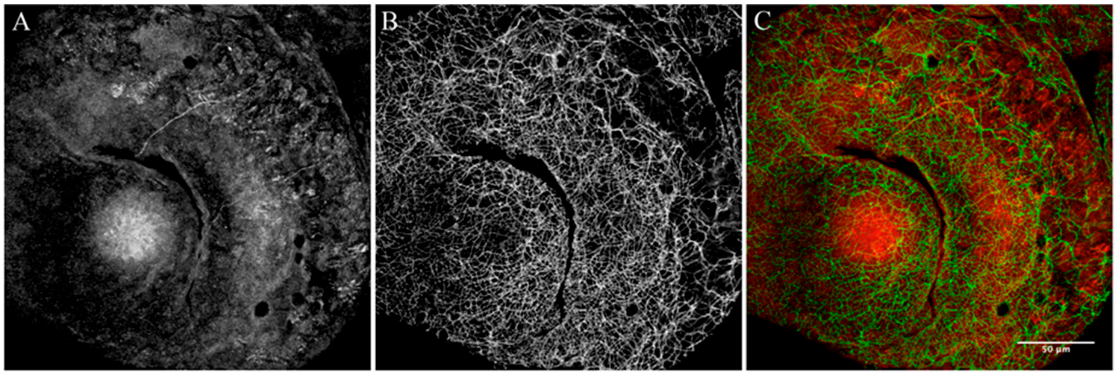

Figure S1. Mmp2 immunoreactive fibers are not Zn-12 positive sensory fibers. Confocal projections of a stack through the eye of a 72 hpf embryo double labeled with anti-zebrafish-Mmp2 (**red**) and Zn-12, which labels sensory fibers in zebrafish (**green**). Mmp2 immunoreactivity (greyscale in A and red in C) is evident in two fibers crossing the retina, whereas Zn-12 immunoreactivity (greyscale in B and green in C) is abundant in the sensory reticulum on the surface of the retina, but absent in the Mmp2-positive fibers. Scale bar = 50 µm.

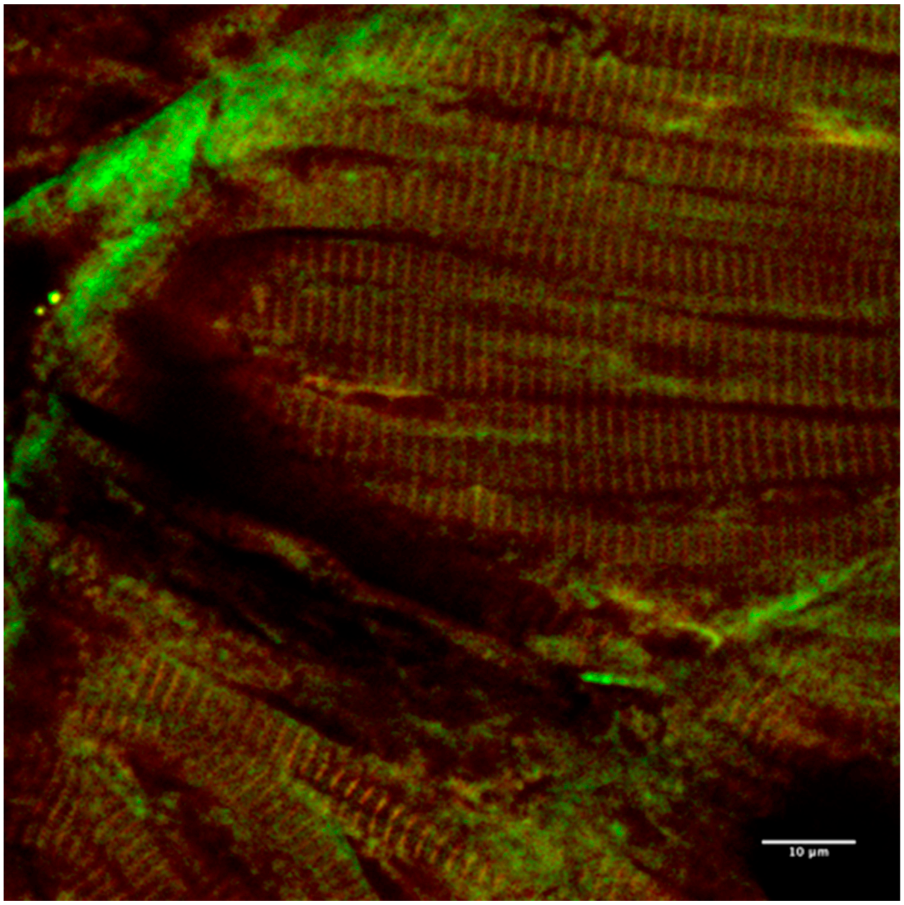

Figure S2. Mmp2 immunoreactity co-localizes with myosin immunoreactivity in skeletal muscle. Confocal section through the trunk musculature of a 72 hpf embryo double labeled with anti-Mmp2 (**green**) and anti-muscle-myosin (F59) (**red**). Where intracellular Mmp2 immunoreactivity is present, it colocalizes with F59 labeling. Scale bar = 10 µm.
